# Synthesis and characterization of maleimide-functionalized polystyrene-SiO_2_/TiO_2_ hybrid nanocomposites by sol–gel process

**DOI:** 10.1186/1556-276X-7-350

**Published:** 2012-06-27

**Authors:** Sivalingam Ramesh, Arumugam Sivasamy, Joo-Hyung Kim

**Affiliations:** 1 Lab of Nano-micro Device, Department of Electronic Engineering, College of Electronics and Information Engineering, Chosun University, Gwangju, 501-759, South Korea; 2 Chemical Engineering Area, CSIR-Central Leather Research Institute, Adyar, Chennai, 600020, India

**Keywords:** Polystyrene maleimide, Optical transparency, Nanocomposites

## Abstract

Maleimide-functionalized polystyrene (PSMA-SiO_2_/TiO_2_) hybrid nanocomposites were prepared by sol–gel reaction starting from tratraethoxysilane (TEOS) and titanium isopropoxide in the solution of polystyrene maleimide in 1,4-dioxane. The hybrid films were obtained by the hydrolysis and polycondensation of TEOS and titanium isopropoxide in maleimide-functionalized polystyrene solution followed by the Michael addition reaction. The transparency of polymer (PSMA-SiO_2_/TiO_2_) hybrid was prepared from polystyrene titanium isopropoxide using the γ-aminopropyltriethoxy silane as crosslinking agent by *in situ* sol–gel process via covalent bonding between the organic–inorganic hybrid nanocomposites. The maleimide-functionalized polystyrene was synthesized by Friedel-Crafts reaction from *N*-choloromethyl maleimide. The FTIR spectroscopy data conformed the occurrence of Michael addition reaction between the pendant maleimide moieties of the styrene and γ-aminopropyltriethoxysilane. The chemical structure and morphology of PSMA-SiO_2_/TiO_2_ hybrid nanocomposites were characterized by FTIR, nuclear magnetic resonance (NMR), ^13^ C NMR, SEM, XRD, and TEM analyses. The results also indicate that the inorganic particles are much smaller in the ternary systems than in the binary systems; the shape of the inorganic particles and compatibility for maleimide-functionalized polystrene and inorganic moieties are varied with the ratio of the inorganic moieties in the hybrids. Furthermore, TGA and DSC results indicate that the thermal stability of maleimide-functionalized polystyrene was enhanced through the incorporation of the inorganic moieties in the hybrid materials.

## Background

Nanostructure hybrid organic–inorganic composites have attracted considerable attention recently, both from the perspectives of fundamental research and their technological applications [1-3]. One approach for preparing these materials is via sol–gel process. In the inorganic matrix, components are formed *in situ* through hydrolysis and condensation of metal oxide precursors, while the organic matrix undergoes simultaneous polymerization. However, the sol–gel approach is limited by the evaluation of volatile biproducts and concomitant shrinkage when the hybrid is processed at elevated temperature [4,5]. The sol–gel method is one of the most suitable methods to prepare the silica gel through siloxane linkages by the hydrolysis and condensation reactions. The silica hybrid materials greatly depend on the interaction between the organic polymers and inorganic alkoxides and their homogeneous distribution through hydrogen bonding, covalent bonding, formation of stereo regular complex, and π-π and ionic interactions [6-8]. The Michael addition reaction in polymer synthesis and applications in emerging technologies, composites, coatings, and optical coatings are outlined in the article [9]. Organic–inorganic hybrid nanocomposites are a new category of high-performance materials which is currently an area that has received extensive interests for other matrix of polyimide (PI)-SiO_2_[10,11], PI-Al_2_O_3_[12], and PI-TiO_2_[13-15]; these composites have been successfully synthesized. Many researches have been focusing on developing the PI-inorganic hybrid nanocomposites, such as the use of dianhydride and diamine to synthesize the PI matrix and the use of metal alkoxides to provide the inorganic network. Nanocomposites can be prepared through different processes. Among those successful ones [16-19], the *in situ* polymerization and gelation reaction is a type of processing in which the inorganic phase was generated from the metal alkoxide precursors through hydrolysis and condensation reactions that took place simultaneously with the polymerization reaction. Wang and Chang and other researchers [20,21] prepared the hybrid nanocomposite films of TiO_2_ in the PI matrix from 2,5-bis (4-aminophenyl)3,4-oxadiazole, 4,4′-oxydiaphthalic anhydride, and titanium precursors by an *in situ* sol–gel process. The titanium precursor was prepared by mixing tetraethyl titanate (TET) and acetyl acetone (acac) in the solution of alcohol and water. These nanocomposites exhibited fairly good optical transparency at 40 wt.% of TiO_2_ content. The transmission electron microscope (TEM) results showed that the size of the TiO_2_ particle increased from 10 to 40 nm [20-22]. Several successful examples of *in situ* polymerization and gelation reaction processes could be found in the literature. The polymer nanocomposites were prepared using poly(amic acid) solution by the condensation of 3,3′,4,4′-benzonphenone tetra carboxylic dianhydride and 4,4′-oxydianhydride 4,4′-oxydianiline (ODA) then added TET followed by thermal imidization from PI/TiO_2_ hybrid nanocomposites. They reported that nanosized inorganic TiO_2_ network dispersed in PI films at an average diameter of 1.5 nm at TiO_2_ content of 12 wt.% [22-24]. The poly(amide-imide)/TiO_2_ (PAI/TiO_2_) nanocomposite films obtained 4,4′-oxy(phenyl trimellimide) and ODA using tert-butyl benzoic acid as the mono functional endcapper; with TET, these composite films exhibited high transparency and had well-dispersed nanosized TiO_2_ in the PAI matrix [25-27]. The size of the TiO_2_ network increased from 5 to 50 nm when the TiO_2_ content was increased from 4% to 18% by weight. While large sized, nanosized inorganic particles made the nanocomposite films, transparent in such the particle size effect, hydrogen bonding between the amide group in the PAI and the hydroxyl groups on the inorganic oxides played an important role in making such small particle size possible [28-30]. According to these reports, the sol–gel process is one of the most commonly used processes of preparing titanium dioxide. However, in the process for preparing titanium dioxide, there are a few technical problems that must be resolved. First of all the titanium alkoxide is a highly reactive compound when it is exposed to moisture, and white precipitate will form rapidly. In order to prepare nanosized TiO_2_ suspension solution, the pH values and the use of chelating agents are crucial in the reaction steps [31]. It is reported elsewhere in the literature that [32-34] prepared high refractive index thin films of pyrometallic dianhydride titania hybrid materials from dianhydride, γ-aminopropyltriethoxy silane (γ-APS), and titanium isopropoxide via sol–gel process followed by spin coating and multi-step backing; through adjustment in the concentration and reaction time, they were able to produce thin films of hybrid inorganic content at 59.2%. Therefore, the transparent polymer hybrid used for lenses has about 90% transmittance/cm. Typical materials PMMA, PC, polystyrene, and styrene-MMA copolymers have been used for the lenses in projection television and lenses for using compact disk. The plastic materials in optical disk circuit need to be highly transparent, resistant to heat, low impurities, and must have a low double refractive index and low fluidity. However, no materials with these properties have yet been found or developed. Semiconductor-mediated photocatalytic oxidation has been accepted as a promising method for the removal of organic contaminants from waste water. Among the semiconductors employed, TiO_2_ is known to be a good photocatalyst because of its high photosensitivity, non-toxicity, easy availability, strong oxidizing power, and long-term stability [35,36]. Existing bulk semiconducting materials possess low surface area, less absorption property, and fast electron–hole recombination. In order to circumvent such problems, researchers are interested in recent days in the synthesis of nanomaterials for environmental applications [34-36]. The polymer metal oxide hybrid nanocomposites contained bimetallic dopants of the titanium and barium oxides. The precursors of metal oxides were formed from tetrabutyl titanate (TBT) and barium carbonate, which were then mixed with poly(amic acid) solution followed by thermal imidization. The synthesized hybrid nanocomposites with inorganic particles are smaller than 50 nm, and the dielectric constant increased from 3.5 to 4.2 when the inorganic content increased from 1 to 10 wt.%. Polyimides are considered to be one of the most important super-engineering materials due to their thermal stability as well as the superior mechanical properties at elevated temperature [37,38]. Since the polyimide/silica hybrid materials have been prepared successfully through the sol–gel process [39], more attention were given to the field [34-40]. Recently, metal-containing polyimide/titania hybrids were also prepared [41]. The key challenge for the preparation of the hybrid materials is how to control the phase separation between the organic and inorganic moieties. The phase behavior is connected with the interaction between the organic segment and the inorganic network in the hybrids. Hydrogen bonding or covalent bonding is usually used to prevent phase separation [42]. Recently, the sol–gel process is a novel technique for the preparation of nanocrystalline TiO_2_. It has been demonstrated that through the sol–gel process, the physico-chemical and electrochemical properties of TiO_2_ can be modified to improve its efficiency. It provides a simple and easy means of synthesizing nanoparticles at ambient temperature under atmospheric pressure, and this technique does require simple setup. Since this method is a solution process, it has all the advantages over other preparation techniques in terms of purity, homogeneity, felicity, and flexibility in introducing dopants in large concentrations, stoichiometry control, ease of processing, and composition control. Through the sol–gel process, the growth of TiO_2_ colloids in sub-micrometer range can be effectively controlled by hydrolysis and condensation of titanium alkoxides in aqueous medium [43]. Nanosize TiO_2_ used so far in photocatalytic applications has been prepared by hydrolysis of titanium precursors followed by annealing, flame synthesis, and hydrothermal and sol–gel processes. In most studies, attempts have been made to enhance the photocatalytic activity of TiO_2_ only by varying the calcination temperature and, in a few cases, aging period and drying conditions [40-43]. Among the various techniques under the development, the sol–gel process has been found to be extremely suitable as it enables good control of composition and optical behavior of the final nanomaterials. In recent years, silica-titania hybrid organic–inorganic materials have been studied as a promising system for photonic applications [41-43], and low loss wave guide based on the organically modified alkoxides has been fabricated by the sol–gel process. Therefore, the sol–gel integrated optics is beginning to show potential applications, and it stimulates the studies on optical wave guide material which have been explored for a long time and such sol–gel materials used for optical applications [40]. In the area of advanced oxidation technology, titanium dioxide semiconductor photocatalysis has been widely studied because of its potential application in air clean-up and water purification. TiO_2_ is largely used as photocatalyst due to its beneficial characteristics: high photocatalytic efficiency, physical and chemical stability, low cost, and low toxicity [40-42]. TiO_2_/SiO_2_ composites are very promising in the field of heterogeneous photocatalysis since they could provide simultaneously enhanced photocatalytic and thermal properties compared to pure TiO_2_ photocatalyst. It has been reported that photocatalytic reactivity of TiO_2_/SiO_2_ nanocomposites is highly dependent on the Ti/Si ratios. The photocatalytic activity and mechanical stability were reported to improve by the addition of about 50% SiO_2_[40-48]. Moreover, in the present work, an attempt has been made to develop the silica/titania-incorporated transparent maleimide-functionalized polystyrene to improve the thermochemical and optical characteristics with γ-APS, TEOS, and titanium isopropoxide through the Michael addition reaction at relatively low temperature through the sol–gel method. Furthermore, the organic–inorganic transparent hybrid nanocomposite materials were characterized by FTIR, nuclear magnetic resonance (NMR), thermogravimetric analysis (TGA), differential scanning calorimeter (DSC), optical images, scanning electron microscope (SEM), and TEM analyses.

## Methods

### Materials

Maleimide, phosphorous trichloride, stannic chloride, γ-APS, TEOS, titanium tetraisopropoxide, and acetylacetone, obtained from Aldrich chemical company (Sigma-Aldrich Corporation, St. Louis, MO, USA), were used as received. Polystyrene obtained from Supreme Petrochemical Ltd, Mumbai, India. The 1,4-dioxane was purified by distillation in vacuum, and other reagents were used as received from SRL, India. FTIR spectra was taken using a PerkinElmer (Model RX1, Waltham, MA, USA) spectrometer (cured samples were ground with solid KBr). ^1^ H (400 MHz) and ^13^ C (200 MHz) NMR spectra were recorded on a JEOL GSX 400 spectrometer (JEOL Ltd., Akishima, Tokyo, Japan) operating at 298 K with tetramethylsilane as an internal standard. Differential scanning calorimetry was performed using both Netzsch (Erich Netzsch GmbH and Co., Bavaria, Germany) and TA instruments (TA 2000 analyzer, New Castle, DE, USA). Cured samples (50 mg) were analyzed in open (silicon) pans at 20 K/min in N_2_ atmosphere. X-ray diffraction analysis (XRD) was characterized by Rigaku Corporation D/max-3 C (Tokyo, Japan). Scanning electron microscopy of fractured surfaces was performed using a JEOL JSM model 6360 microscope. The fractured surfaces of the specimens were coated with platinum and were exposed to accelerating voltage of 20 kV. The particles in solution are characterized by placing a drop of the homogeneous suspension in a TEM copper grid with a lacy carbon film and then using a JEOL 2010-F TEM at an accelerating voltage of 200 kV.

### Synthesis of *N*-chloromethyl maleimide

**Figure 1:**
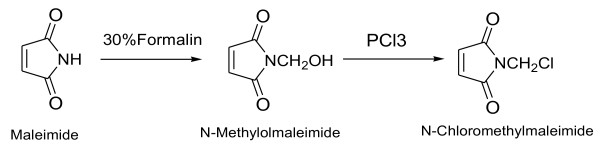
**Synthesis of *N *-chloromethyl maleimide.**

*N*-chloromethyl maleimide was prepared in two steps [1-4]. In the first step, *N*-ethylmaleimide was prepared using a suspension of 24.5 g (0.25 mol) of maleimide in 20.3 ml of 37% formalin at 30°C with the addition of 0.75 ml of 5% NaOH over a period of 30 min and allowing it to stand for 3 h and then filtered. The crude product yield of 75%, m.p = 103°C, was recrystallized using ethyl acetate ^1^ H NMR (CDCl_3_), *δ* (ppm) = 3.45 (S, 1 H), 5.09 (S, 2 H), and 6.76 (S, 2 H) and ^13^ C NMR, *δ* (ppm) = 61.11, 134.71, and 70.25 (C = O). In the second step, phosphorous trichloride of 4.3 g (0.03 mol) was added to the solution of 10 g (0.08 mol) of *N*-methyl maleimide in 50 ml of acetone in an ice bath. Then, the solution was stirred for 30 min and then concentrated at the aspirator. The resulting partly crystalline residue was precipitated by adding 50 ml of ice-cold water and filtered. The product *N*-chloromethyl maleimide was recrystallization from benzene-petroleum ether mixture. The sequence of reaction *N*-chloromethyl maleimide is presented in Figure 1^1^HNMR (CDCl_3_) *δ* (ppm) = 5.32 (S, 2 H), and 6.84 (S, 2 H); ^13^ C NMR *δ* (ppm) = 44.28, 135.27, and 168.17 (C = O).

### Synthesis of maleimide-functionalized polystyrene

**Figure 2:**
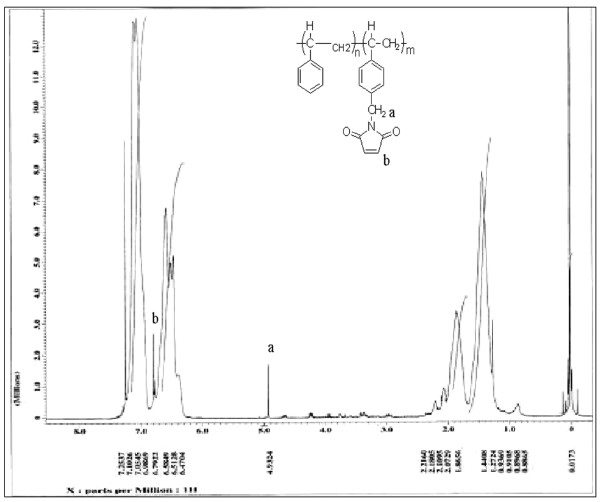
^**1**^**H NMR spectrum of maleimide-functionalized polystyrene.**

**Figure 3:**
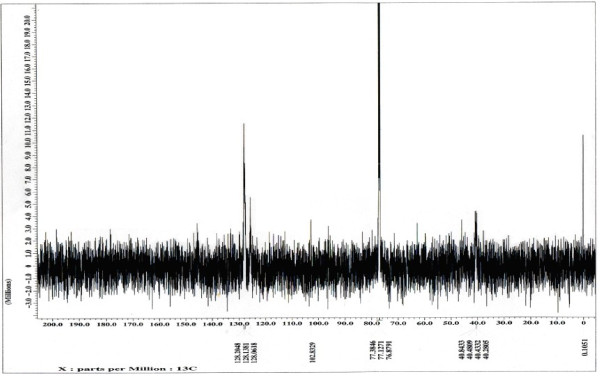
^**12**^** C NMR spectrum of maleimide-substituted polystyrene.**

**Figure 4:**
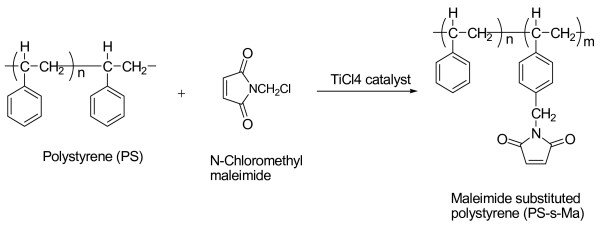
Synthesis of maleimide-functionalized polystyrene.

Maleimide groups were functionalized into polystyrene by a mild Friedel-Crafts process using *N*-chloromethyl maleimide following the reported procedure [1-4]. The 5.20-g polystyrene (50 mmol of repeat unit) dissolved in 25 ml of 0.1 M solution of chloroform was completely clear and allowed to left under nitrogen atmosphere. The mixture was stirred until the solution was completely clear and allowed to left under nitrogen atmosphere for 24 h with constant stirring. Then, the resulting polymer (maleimide-functionalized polystyrene (PSMA)) was precipitated in excess of methanol and filtered, redissolved, and reprecipitated twice and dried in a vacuum oven at 50°C to get constant weight. The mild reaction condition procedure was adopted to avoid any side reactions during maleimide methylation of polystyrene. The maleimide functionalized in polystyrene was confirmed, and the percentage substitution was calculated using ^1^ H NMR and ^13^ C NMR as presented in Figures 2 and 3. The sequence of reaction involved in the synthesis of maleimide-substituted polystyrene is given in Figure 4. ^1^HNMR − *δ* (ppm) = 1.2 to 2.2 (broad, aliphatic protons), 4.93 (S-CH_2_, protons), 6.4 to 7.5 CH_2_ (broad, aromatic protons), and 6.79 (S, maleimide protons); ^13^CNMR 40.8, 41 (CH_2_), 168 (C = O) ,135.2 (C = C), 138.5 (C-N), and 168.17 (C = O).

### Maleimide-substituted polystyrene-silica-titania hybrid nanocomposites

#### Preparation of the hybrid films

**Figure 5:**
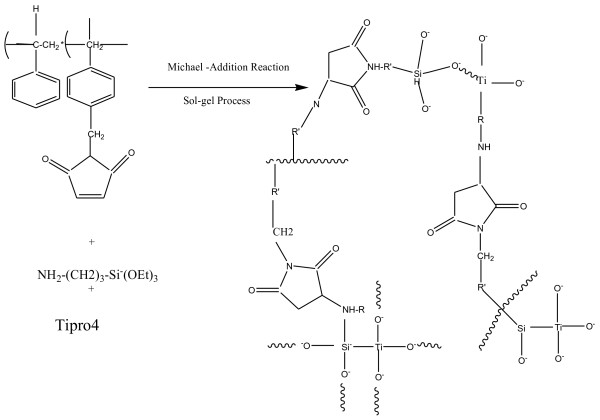
**Formation of PSMA and γ-APS-SiO**_**2**_**/TiO**_**2**_** hybrid nanocomposites by sol–gel reaction.**

Polyimide is insoluble in organic solvents. The sol–gel reaction was carried out in the solution of polyamic acid, and the poly(amic acid) (PAA) and HCl work as the acid catalysts of the hydrolysis. After the hydrolysis was carried out for 6 h, the resultant homogeneous mixture solution was cast on to the glass plate to prepare PAA hybrid film. The PAA hybrid film was obtained by drying the cast film at 80°C to evaporate the solvent, unreacted TEOS (and TBT), ethanol, water, etc. In the process of drying, the siloxane network of silica between PAA and TEOS molecules and among TEOS molecules was developed [17]; for the titania systems, the network between PAA and TBT molecules and among TBT molecules may be developed, particularly, in the ternary hybrids of polyimide/silica-titania systems. A network between TEOS and TBT molecules may also be expected. The PI hybrid film was obtained by thermally treating the precursor film at higher temperature. The polymer hybrids (Figure 5) were synthesized through the sol–gel process (Table 1) using PSMA and γ-APS in tetrahydrofuran (THF) as the solvent. To the PSMA solution, γ-APS was added dropwise with constant agitation in the presence of hydrochloric acid (0.01-M HCl aqueous). Then, after 30 min, the calculated quantities of acac and titanium isopropoxide were added into the polymeric matrix with vigorous stirring for 12 hours. The resulting mixture was poured into a polypropylene container, the solvent was allowed to evaporate, then the glassy polymer hybrid film was obtained after 15 h. The resulting films were cured and dried then subjected to further analysis. The hybrid films of both PI/SiO_2_ and PI/TiO_2_ were transparent in which the inorganic content is lower than 5 wt.%, semitransparent when the inorganic content is 8 wt.%, and opaque when the inorganic content is beyond 10 wt.%. The ternary hybrids films of PI/SiO_2_-TiO_2_ were prepared with varied inorganic (SiO_2_-TiO_2_) contents, and three serious ternary hybrids were synthesized in the ratio of SiO_2_/TiO_2_ at 2:1, 1:2, and 1:1 by weight, respectively. The transparent PAA/SiO_2_-TiO_2_ solution was obtained when the inorganic portion is no more than 30 wt.% for the SiO_2_/TiO_2_ (2:1) and SiO_2_/TiO_2_ (1:2) systems, while 50 wt.% for the SiO_2_/TiO_2_ (1:1) systems [35-40].

**Table 1:** Experimental details of organic–inorganic polymer hybrid sol–gel nanocomposites

**Experiment**	**PSMA** **(g)**	**γ-APS** **(ml)**	**Ti(ipro**_**4**_**)** **(ml)**	**Solvent** **1,4-dioxane (ml)**	**0.1-M HCl in aqueous**	**Temperature (°C)**	**Appearance**
PS-Ti-a	0.050	-	0.050	2	0.0012	40	Turbid
PS-Ti-b	0.050	0.0083	0.040	2	0.0150	60	Transparent
PS-Ti-c	0.025	0.0083	0.0125	2	0.0140	60	Transparent
PS-Ti-d	0.010	0.0041	0.0050	2	0.0070	90	Transparent
PS-Ti-e	0.005	0.0020	0.0025	2	0.0035	50	Translucent

## Results and discussion

### Preparation of organi-inorganic hybrid nanocomposites controlled by Michael addition reaction

In the present study, the PSMA-γ-APS and TEOS were used to prepare the transparent polymer hybrid PSMA which was synthesized by mild, Friedel-Crafts reaction between the *N*-chloromethyl maleimide and polystyrene in the presence of stannic chloride or TiCl_4_ as a catalyst. The amount of maleimide substitution in polystyrene depends on the percentage weight of *N*-chloromethyl maleimide added to the polystyrene. The maximum time required to achieve 16% of maleimide substitution on polystyrene is about 20 h. The percentage maleimide substitution was calculated using the integration values obtained from ^1^ H NMR spectrum. Figure 1 illustrates the sequence of reaction involved in the preparation of transparent organic–inorganic polystyrene maleimide silica hybrid using γ-APS as cross linking agent by Michael addition reaction through *in situ* sol–gel process. Table 1 summarizes the composition of reactants for the preparation of hybrid polymer. In order to achieve the homogeneity and transparency of the resulting hybrid polymer, suitable experimental conditions have been adopted by adjusting the concentration of both TEOS and γ-APS in the reactions. It was ascertained that the experiments conducted in the absence of γ-APS result in only turbid materials, while the homogeneous polymer hybrids were obtained in the presence of γ-APS as per experiments in Table 1 which shows the optical and SEM images of the polystyrene-silica hybrids. In case of the absence of γ-APS, phase separation was clearly observed, and the silica gel domain was larger than 5 μm. On the other hand, no phase separation was observed when the experiment was carried out using γ-APS [30-35]

### Control of the SiO_2_/TiO_2_ nanoparticle in the PSMA matrix

The silicic acid was prepared according to the procedure described previously [23]. A silicic acid THF solution and a TiCl_4_ ethanol solution were mixed and stirred at room temperature for 3 h. The mole ratio of silica to titania was 1. Appropriate amounts of silica/titania precursor were introduced into a THF solution of poly (MMA-co-MSMA) copolymer, and the mixture was stirred mechanically at room temperature for 4 h to obtain a uniform and transparent solution. The solution was cast on a glass substrate and dried at 80°C for 5 h. Then, the samples were thermally treated at 100°C for 10 h under vacuum after drying the resulting mixture for the preparation of PMMA/silica/titania nano composites [20-25]. In this study, synthesis and characterization of polystyrene maleimide-SiO_2_/TiO_2_ hybrid nanocomposites with the formation of covalent bonding between the siloxane segment of γ-APS containing PSMA matrix and the network through the introduction of the coupling agent (γ-APS) were done. On the basis, it was expected that these interfacial covalent bonds would render the material's different properties, which will be discussed shortly. For the example, large solubility parameter difference between PI and PDMS interfacial gap that existed will lead to micro-phase separation to minimize the interfacial gap; the titanium alkoxide, such as titanium (IV) tetra ethoxide Ti(OEt)_4_, TET, titanium isopropoxide (Ti(ipro_4_)), is generally used as the starting material of TiO_2_ in the sol–gel process [12-14,30-36].

### FTIR spectra of the obtained polymer hybrids

**Figure 6:**
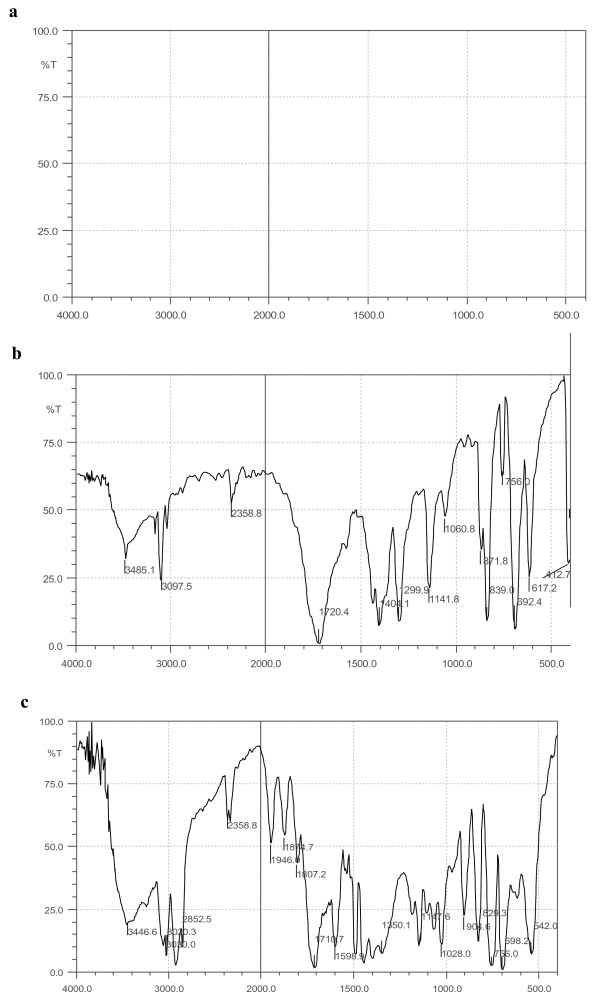
**FTIR spectra.** (**a**) *N*-methylolmaleimide, (**b**) *N*-chloromethyl maleimide, and (**c**) maleimide-functionalized polystyrene.

The Michael addition reaction involved in the polymer hybrid formation was confirmed by FTIR. Figure 6a,b,c shows the FTIR spectra of *N*-chloromethyl maleimide (MA-Cl) and polystyrene-functionalized maleimide (PS-s-MA); it is observed that the peak corresponds to the maleimide group at 3,096,830 cm^−1^ of H-C = C-H stretching and at 1,702 and 1,725 cm^−1^ for C = O stretching seen in all the cases which confirm the maleimide substitution without ring opening of maleimide. Figure 6a,b presents the FTIR spectra of γ-APS, PS-s-MA, and γ-APS and that of the homogeneous polymer hybrids. In the case of γ-APS, only a broad peak at 3,350 cm^−1^ for primary amino group was obtained, whereas for PS-s-MA, there is a peak at 3,100 cm^−1^ for maleimide H-C = C-H stretching and at 1,725 cm^−1^ for C = O stretching observed. The disappearance of peaks corresponding to C = C of maleimide at 3,100 and at 830 cm^−1^ which diminishes the appearance of secondary amino group after Michael addition reaction was ascertained from the IR spectra as shown in Figure 6c. The disappearance of maleimide double bond at 3,100 cm^−1^ confirms the occurrence of Michael addition reaction during the formation of PS-s-MA-silica hybrid; these results are in good agreement with the SEM micrographs obtained for the homogeneous transparent polymer hybrids. The bands at around 650 and 1,100 cm^−1^ are representative of TiO_2_ and SiO_2_ matrixes in nanocomposite. The band at around 950 cm^−1^ has been assigned to the stretching of the Si-O species of Si-O-Ti or Si-O defect sites which are formed by the inclusion of Ti^4+^ ions into the SiO_2_ matrixes. Thus, the appearance of the band at around 950 cm^−1^ indicates that the TiO_2_ species are embedded into SiO_2_ matrixes within the TiO_2_/SiO_2_ nanocomposite. The broad peak appearing at 3,100 to 3,600 cm^−1^ is assigned to the fundamental stretching vibration of hydroxyl groups (free or bonded) which is further confirmed by the weak band at about 1,620 cm^−1^[20-23]. FT-IR spectrum of the as-synthesized composite has three characteristic bands that appeared at around 1,100, 950, and 650 cm^−1^. The bands at around 650 and 1,100 cm^−1^ are representative of TiO_2_ and SiO_2_ matrixes in nanocomposite.

### Differential scanning calorimeter

**Figure 7:**
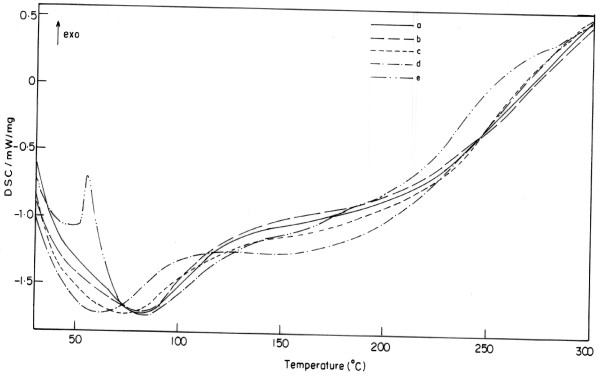
**DSC thermograms (a to e) of PSMA-SiO**_**2**_**/TiO**_**2**_** hybrid nanocomposites.**

The DSC thermograms of PSMA, PSMA and γ-APS, and homogeneous transparent polymer hybrids are presented in [1,4,35-40] (figures were not shown). From the thermograms, it is observed that the glass transition temperatures of unmodified polystyrene and maleimide-modified polystyrene (PSMA) are 86°C and 92°C, respectively. Functionalized polystyrene has higher glass transition temperature (*T*g) values than that of unmodified polystyrene. Then, the incorporation of γ-APS to the functionalized polystyrene (PSMA) further enhanced the value of *T*g to 95°C which is higher than that of the functionalized polystyrene. The increase in *T*g values may be explained due to the Michael addition reaction which induces to form the cross-links between the organic polymer and the silica gel, hybrid nanocomposites. The incorporation of TiO_2_ into functionalized hybrid materials (PSMA) enhances the thermal stability of the resulting polymer hybrid nanocomposites. The concentrations of TiO_2_ in the samples designated as PSMA-Si-Ti a, b, c, d, and e are presented in Table 1 and Figure 7. From the results, the *T*g of the hybrid nanocomposites a, b, c, d, and e are 151°C, 167.4°C, 174.5°C, 123.2°C, and 103.3°C, respectively. Upon decreasing the weight percentage of TiO_2_, the values decreased due to the effect of occurring cross-linking reactions to a maximum extent and the change of phase imparted by the metal oxide. The interaction between the silica-titania hybrid polymer matrix resulted from the self condensation of SiOH or TiOH generated by the hydrolysis which resulted in a decrease in the molecular mobility and increase in the *T*g for organic–inorganic hybrid nanocomposites. Particularly, the concentration of the PSMA/TiO_2_ chains is seriously restricted by the silica/titania linkages. The restriction could be also coming from the cross-linking ability and interphase bonding changes for metal oxide and organic matrix [11-14].

### Thermogravimetric analysis

**Figure 8:**
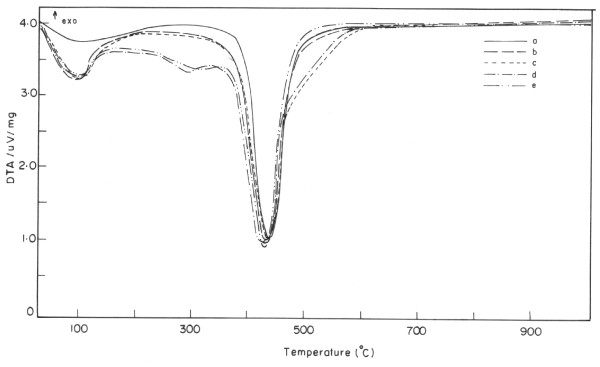
**DTA curves (a to e) of PSMA/TiO**_**2**_** at various concentrations of hybrid nanocomposites.**

**Figure 9:**
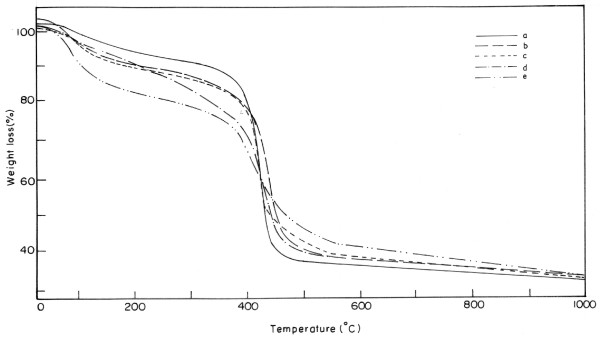
**TGA curves (a to e ) of PSMA-SiO**_**2**_**/TiO**_**2**_** hybrid nanocomposites.**

Thermal stability of the PSMA/TiO_2_ hybrid was ascertained using TGA [1-10]. The TGA and DTA thermogram homogenous transparent hybrids are presented in Figures 8 and 9. The presence of inorganic content, i.e., 30% in the polymer hybrid was established from the TGA, which is much lower than that of theoretical value, i.e., 35%. This may be due to the presence of residual silanol or >Ti-OH groups or unreacted alkoxy groups or the secondary amino group left after Michael addition reaction that remains in the polymer hybrids, which leads to mass loss at lower temperature (200°C). However, in the present study, it was observed that there is an increase in thermal stability of the polymer hybrid due to the strong interactions that resulted between the metal oxide and polymer matrix. The initial degradation temperature of the transparent hybrid was lower than that of the functionalized polystyrene, and the decrease in thermal stability was due to the presence of γ-APS in the hybrid. Its decomposition starts about 210°C. In addition, the presence of unreacted alkoxy groups and weak amine linkages imparted by Michael addition reaction during the formation of polymer hybrid also causes the lower initial degradation temperature. The thermal stability data from TGA and DTA show that degradation temperature was found to be in two stages. This may be due to the presence of residual SiOH or TiOH, unreacted alkoxy groups, or the secondary amino group left after Michael addition that remains in the polymer hybrids. For the first and second stages, the degradation temperatures for each concentration are shown in Table 2. The thermal stability for hybrid nanocomposites at high temperature exceeds that of functionalized polystyrene which suggests the successful incorporation of the silica/titania moiety through chemical interactions into the polymer matrix. Furthermore, it is ascertained that the rigid siloxane and titania particles are surrounded by the organic polymer at molecular level interaction through hybridization, which imparts higher thermal stability for hybrid nanocomposites. The formations of organic–inorganic hybrid nanocomposites at molecular level dispersion is also ascertained from SEM studies in Figures 7 and 8.

**Table 2:** **Thermal properties of organic–inorganic PSMA-SiO**_**2**_**/TiO**_**2**_** hybrid sol–gel nanocomposites**

**System**	***T*g (**°C**)**	**First degradation temperature (**°C**)**	**Second degradation temperature (**°C**)**	**Characteristic yield at 999.1** °C **(**%**)**
PSMA-Ti-a	151.0	430.8	589.7	34.40
PSMA-Ti-b	167.4	440.7	603.9	22.60
PSMA-Ti-c	174.5	442.2	604.3	38.26
PSMA-Ti-d	123.2	425.8	600.0	43.75
PSMA-Ti-e	103.3	422.7	597.3	44.19

### Optical transparency of SiO_2_/TiO_2_ hybrid nanocomposites

**Figure 10:**
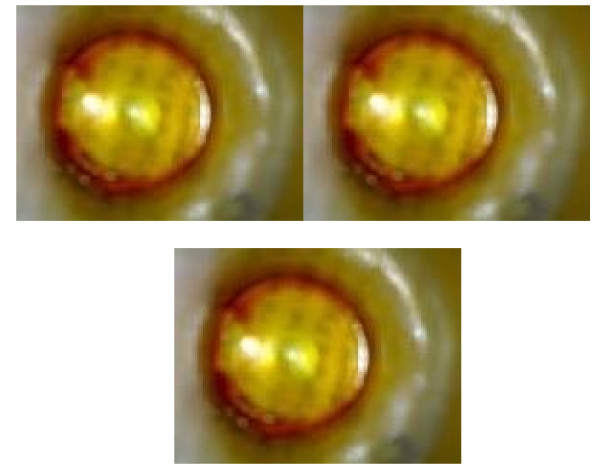
**Optical images of the polystyrene maleimide-SiO**_**2**_**/TiO**_**2 **_**hybrid nanocomposites.**

The optical transmittance of the hybrid coating films has different TEOS molar content. Light transmittance increased with increasing of TEOS content up to 0.04 mol in all visible light range of 400 to 800 nm. In particular, at 0.04 mol of TEOS content, a coating film with enhanced optical transparency could be obtained. This result may be explained by the fact that the addition of TEOS precursor up to some level of content results in more homogeneous and finer microstructure in the hybrids due to increased interfacial attraction between two phases. However, in the case of hybrid coating films with addition of 0.07- and 0.10-mol TEOS, the optical transparency was reduced compared with the coating film with addition of 0.04-mol TEOS. It can be believed that at above 0.04-mol TEOS content, the decrease in light transmittance with the increase of TEOS content may result from the formation of silica clusters during elation of the hybrids followed from the occurrence of micro-phase separation between organic and inorganic phase, which can be seen that inorganic silica is involved in the hybrid; micro-cracks can be easily formed on the surface of the coating film due to reduced flexibility, so these defects increase light scattering on the film. This can be evidenced by surface morphology, which was observed by SEM, for coating films with various TEOS contents [30,35]. It should be noted that there existed no micro-crack on the surface of the hybrid coating film obtained from the addition of 0.04-mol TEOS content; on the other hand, at above 0.04-mol TEOS content, the degree of micro-crack formation increased with increase of TEOS content. From these results, the optimal content of inorganic silica precursor, TEOS, was found to be 0.04 mol of PVA/SiO_2_ hybrids with enhanced microstructure and optical transparency of the hybrid materials [30,35,41]. The hybrid nanocomposites exhibit an optically transparent behavior which is shown in Figure 10 and Table 1. It is observed that the formation of hybrid materials is in turbid form in the absence of γ-APS. However, in the presence of γ-APS, the homogeneous polymer hybrids were obtained. The phase separation of the hybrid was noticed if the silica/titania domain was larger than 5 μm. The Michael addition [1-4] reaction proceeds with the pendant maleimide-substituted polystyrene, and the ethoxy groups present in γ-APS undergo hydrolysis followed by condensation reaction with the growing siloxane/titania network which leads to form the hybrid particles less than 5 μm. The optical properties of hybrid nanocomposites were also correlated with the results of SEM analysis. The SEM image of the polymer hybrid prepared from varying composition of PSMA/titanium isopropoxide (in acetyl acetone condition), confirms the nanometer level integration of organic–inorganic hybrid formation in the presence of an γ-APS. This may lead to the fact that a low temperature at 40°C, the rate of Michael addition is low when compared to the hydrolysis and condensation reaction of γ-APS and titanium isopropoxide (TiO_2_) which in turn leads to an increase in the particle size of siloxane and titania network. Whereas, in case of temperature above 60°C, both the reactions occur in equal rate to reduce particle size of both organic and inorganic domains [35-40].

### X-ray diffraction analysis

**Figure 11:**
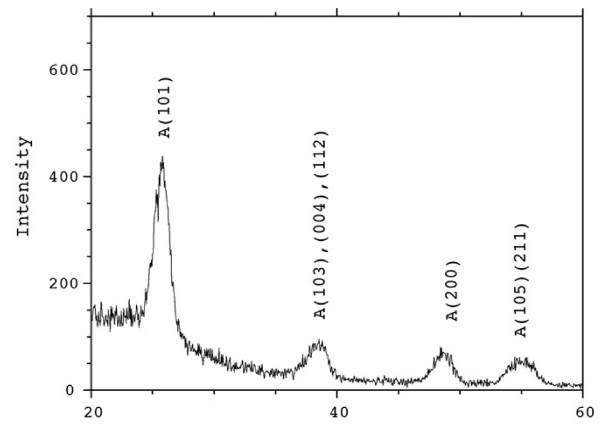
**X-ray diffraction analysis of polystyrene maleimide-SiO**_**2**_**/TiO**_**2 **_**hybrid nanocomposites.**

XRD analysis of synthesized composite and calcined samples reveals that as-synthesized TiO_2_/SiO_2_ nanocomposite has crystalline anatase phase in amorphous silica matrix (Figure 11). Both calcined nanocomposites TS800 and TS1100 have anatase phase TiO_2_, but in TS1100, amorphous silica transforms to crystobalite silica phase. Both the interactions of Si-O-Ti and high dispersion of TiO_2_ in SiO_2_ prevent the crystalline transition to rutile [24,25]. The sizes of the anatase crystallites in the prepared TiO_2_/SiO_2_ nanocomposite samples measured according to the Scherrer equation are 5.0, 7.8, and 26.7 nm for RSR, TS800, and TS1100, respectively. Doping of SiO_2_ into TiO_2_ could effectively retard the growth of nanoparticles and, thus, reduce the particle size. This observation may have resulted from the formation of the Ti-O-Si bond and due to the presence of amorphous SiO_2_ around TiO_2_, which would prevent the growth of TiO_2_ particles [26]. The particle size of TSR and TS800 is close together, but at 1,100°C, a clear jump in the particle size is seen due to the transformation of the particle size which is due to the transformation of amorphous silica to crystobalite [27-30]. The TEM images of the resultant nanohybrid particles obtained under the super critical drying conditions mentioned [30-33] exhibited that almost all the nano-crystals of TiO_2_ existed on the monodispersed SiO_2_ without coagulation. We also have confirmed the same growth plane of (1 1 2) in our previous paper for the TiO_2_-SiO_2_ nanohybrid particles prepared by the conventional drying process [30-34]. Next, we discuss about the surface chemical bonding of the obtained hybrid particles by estimating the acidic property, which can be determined by FTIR and XRD studies of adsorbed organic materials [30-35].

### Scanning electron' microscope

**Figure 12:**
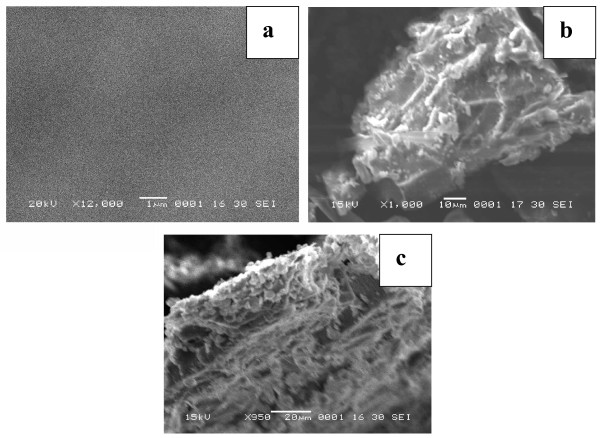
**SEM micrographs.** (**a**) PS MA, (**b**) PSMA + TiO_2_, and (**c**) PSMA + γ-APS-TiO_2_ hybrid nanocomposites.

The sol gel process is used to produce silicon-based polymeric structures as interpenetrating networks in organic binder systems. Conditions for eligibility to participate in an interpenetrated nanocomposite are as follows: the two polymers have to be synthesized in the presence of each other, the two monomers should have the similar reaction kinetics, and the resulting materials should not to be phase separated. Interpenetrated polymer network synthesized in this work was composed of an organic phase (diglycidyl ether of bisphenol), an aromatic amine like HY850, and an inorganic silica phase formed by sol–gel process from TEOS and was coated on the aluminum alloy substrate by dip-coating method. The hybrid network obtained in this manner has an excellent optical transparency and was characterized using different spectroscopic and microscopic techniques. A significant feature in the formation of this hybrid network to enhance compatibility with hybrid materials is the formation of covalent bonding between organic polymers and inorganic compounds. The films on the glass substrates were prepared by dip-coating process from a sol-containing alcoholic tetrabutyl titanate which, after the curing treatment, the gel forms a stable thin homogeneous nanocomposite coating. The films obtained were transparent to the visible light, and their surface hydrophilicity values were increased by increasing TiO_2_ nanoparticle content. Characterization of the nanocomposite coating films performed by TEM showed that the particle size of the superposed TiO_2_ nanoparticles in nanocomposite films were estimated about 2 to 4 nm. Atomic force microscopy observation showed uniformity and three-dimensional surface profile of TiO_2_ nanospheres in the nanocomposite films. The contact angle test without coating and after coating gave a good evidence for hydrophilicity of the prepared nanocomposite coatings and the strong interaction between organic and inorganic phase with the formation of titania domains in the nanoscale range. Applied humidity resistance test showed the high stability of nanocomposite coating [30-35]. The polystyrene maleimide transparent hybrid and turbid products show that the absence of γ-APS, i.e., PS-s-MA and γ-APS, and titanium isopropoxide using acid catalyst results a turbid phase-separated hybrid with titania domains of the size greater than 5 μm. The transparent hybrid shows a clear dispersion of the organic polymer into the titania matrix, i.e., molecular level dispersion is clearly seen in Figure 12. SEM image of domains is seen in the micrograph, which confirms the molecular level dispersion into the matrix. The polymer hybrid prepared in the absence of γ-APS shows that the phase separated the turbid coarse product due to the non-occurrence of Michael addition reaction, whereas the transparent homogeneous polymer hybrid with particle size less than 5 μm, when γ-APS is incorporated along with PS-s-MA-titanium tetra isopropoxide, is better due to the effective combination of Michael addition reaction via the sol–gel process. SEM micrograph of transparent hybrid polymer taken at 1-μm magnification indicates no separate phase domains in the matrix hybrid nanocomposite system [30-38].

### Transmission electron microscope

**Figure 13:**
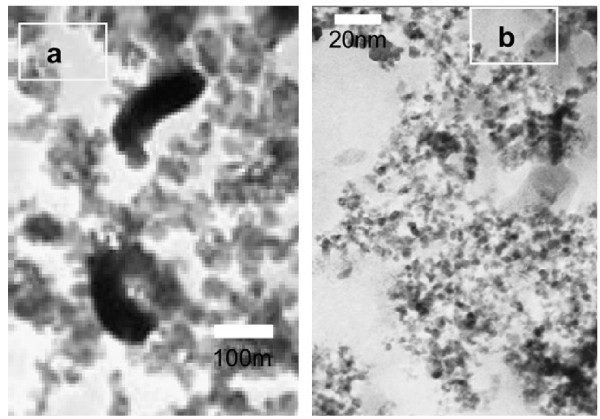
**TEM micrographs of PSMA-γ-APS-TiO**_**2 **_**hybrid nanocomposites.** (**a**) Low and (**b**) high magnifications.

Transparent polystyrene maleimide silica/titania nanocomposites were prepared as transparent polymer hybrid in sol–gel method as shown in Figure 13. TEM results indicate that PSMA and γ-APS and titanium isopropoxide using acid catalyst, which results a turbid phase-separated composite with silica/titania domains of the size, are greater than 5 μm. The polymer and metal oxide is clearly dispersed, and homogeneous structure, i.e., the molecular level dispersion is very clearly seen on the surface of the organic polymer. The polymer hybrid prepared in the absence of γ-APS shows that the phase separated the turbid coarse product due to the non-possibility of Michael addition reaction, whereas the transparent homogeneous polymer hybrid with particle size less than 50 nm, when γ-APS is incorporated along with PSMA-SiO_2_/TiO_2_, is a more effective combination of Michael addition reaction via sol–gel process. The intension of using coupling agent was twofold to minimize the degree of aggregation for an evenly distributed TiO_2_ network and to enhance the organic–inorganic interfacial cohesiveness. The intended role of the coupling agent used (γ-APS) in this study would be no more than proposing a new strategy (type of coupling agent is not limited to γ-APS) in designing this category of the materials with the desired properties for various applications.

## Conclusion

In this study, the PSMA/TiO_2_ hybrid nanocomposite films with covalent bonding at the organic–inorganic interface have been successfully synthesized by sol–gel process. The chelating agent acetyl acetone was used to reduce the gelation rate of titanium alkoxide. The FTIR, optical transparency, and surface morphology analysis confirm the Si-O-Ti and Ti-O-Ti bondings and provided the evidence of the presence of TiO_2_ in the PSMA matrix. The PSMA/TiO_2_ hybrid nanocomposites have good transparency even at high content (up to 30%); therefore, the maleimide-functionalized polystyrene-titania-incorporated nanocomposites have been prepared by Michael addition reaction through the sol–gel process using γ-APS as a cross-linking agent. The polymer hybrid shows optical transparency and improved thermal stability compared with those of functionalized polystyrene. In the present investigation, an attempt has been made to develop hybrid nanocomposites with improved optical transparency and thermal stability through controlled hydrolysis of metal alkoxides. The hybrid materials developed with present expected materials to find the applications in optoelectronics, solar cells, and hybrid coatings for performance and longevity.

## Competing interests

The authors declare that they have no competing interests.

## Authors’ contributions

SR and AS designed, analysed, and performed the experiments and wrote this report. JHK is responsible for the correction of this report. All authors read and approved the final manuscript.
